# Exploring Artificial Intelligence Biases in Predictive Models for Cancer Diagnosis

**DOI:** 10.3390/cancers17030407

**Published:** 2025-01-26

**Authors:** Aref Smiley, C. Mahony Reategui-Rivera, David Villarreal-Zegarra, Stefan Escobar-Agreda, Joseph Finkelstein

**Affiliations:** 1 Department of Biomedical Informatics, University of Utah, Salt Lake City, UT 84108, USA; mahony.reategui@utah.edu (C.M.R.-R.); david.villarreal@utah.edu (D.V.-Z.); joseph.finkelstein@utah.edu (J.F.); 2 Telehealth Unit, Universidad Nacional Mayor de San Marcos, Lima 15081, Peru; stefan1090ea@gmail.com

**Keywords:** cancer, artificial intelligence, bias

## Abstract

The American Society of Clinical Oncology (ASCO) has released the principles for the responsible use of artificial intelligence (AI) in oncology emphasizing fairness, accountability, oversight, equity, and transparency. However, the extent to which these principles are followed is unknown. The goal of this study was to assess the presence of biases and the quality of studies on AI models according to the ASCO principles and examine their potential impact through citation analysis and subsequent research applications. A review of original research articles centered on the evaluation of predictive models for cancer diagnosis published in the ASCO journal dedicated to informatics and data science in clinical oncology was conducted. Seventeen potential bias criteria were used to evaluate the sources of bias in the studies, aligned with the ASCO’s principles for responsible AI use in oncology. The CREMLS checklist was applied to assess the study quality, focusing on the reporting standards, and the performance metrics along with citation counts of the included studies were analyzed. Nine studies were included. The most common biases were environmental and life-course bias, contextual bias, provider expertise bias, and implicit bias. Among the ASCO principles, the least adhered to were transparency, oversight and privacy, and human-centered AI application. Only 22% of the studies provided access to their data. The CREMLS checklist revealed the deficiencies in methodology and evaluation reporting. Most studies reported performance metrics within moderate to high ranges. Additionally, two studies were replicated in the subsequent research. In conclusion, most studies exhibited various types of bias, reporting deficiencies, and failure to adhere to the principles for responsible AI use in oncology, limiting their applicability and reproducibility. Greater transparency, data accessibility, and compliance with international guidelines are recommended to improve the reliability of AI-based research in oncology.

## Simple Summary

Our study examines the use of artificial intelligence (AI) in cancer diagnosis by evaluating the biases and quality of studies published in a prominent oncology journal. The objective is to identify common biases, assess the adherence to the established ethical principles for AI use in oncology, and analyze the impact of these studies on the subsequent research. The findings reveal various biases, including implicit and environmental biases, alongside challenges related to data accessibility and methodological reporting. Consequently, our study highlights the need to conduct methodologically robust research and improve the manuscript reporting practices to enhance the reliability and applicability of AI models in oncology.

## 1. Introduction

Artificial intelligence (AI) models have represented a significant advancement in the screening and diagnosis of various types of cancer, developing models with high levels of sensitivity and specificity [1,2,3], which could potentially improve patient screening and diagnosis. In recent years, significant advances have been achieved in AI applications in cancer diagnostics [4,5,6]. However, the growing development of these tools has raised concerns about the quality of the studies, the bias in the reported results, and the completeness of reporting these models in both hospital and community settings. A meta-review of fifty systematic reviews, including 1100 primary diagnostic accuracy studies of AI, found that most studies had incomplete reporting [7]. Additionally, more than half (57.5%) of all systematic reviews reported a high or unclear risk of bias in the patient selection domain, and one in four (26%) reported a high or unclear risk of bias in the AI performance metrics domain [7]. Moreover, inadequate study quality and incomplete reporting are barriers to the clinical implementation of these technologies and limit the replicability of the studies.

Another potential barrier to the effective implementation and adoption of AI models in oncology is the presence of biases that can compromise the equity and applicability of their outcomes. Specifically, biases such as research bias, provider expertise bias, embedded data bias, environmental and life-course bias, and empathy or contextual bias represent potential risks within the context of AI models for cancer [8]. On the other hand, other types of biases are general across different AI models in healthcare, including implicit bias, selection bias, measurement bias, confounding bias, algorithmic bias, and temporal bias [9]. These biases in studies can lead to less accurate diagnoses or screenings for certain patient groups, affecting their care and clinical outcomes. Hence, it is crucial for research teams developing AI models in oncology to implement bias mitigation strategies, thereby promoting fairer and more inclusive models applicable to diverse contexts and populations.

In addition to addressing bias, it is equally important to adhere to the minimum standards and principles that guide the ethical use and implementation of these AI models. The American Society of Clinical Oncology (ASCO) has established six fundamental principles for the responsible use of AI in oncology, aimed at protecting the ethics and quality of healthcare [10]. In addition, the World Health Organization (WHO) has published guidelines on the ethics and governance of AI in healthcare, within which it proposes six core principles to protect autonomy and promote human well-being [11]. These guidelines complement the ASCO’s principles, providing a comprehensive ethical framework that reinforces the responsible implementation of AI in healthcare.

Although there are principles that promote transparency in studies using AI for cancer diagnosis, such as those established by the ASCO, it remains unclear whether the studies evaluating AI models for cancer diagnosis and screening have reported the minimum information required by these principles. It is also uncertain which types of biases are most prevalent in these studies and whether they present adequate reporting quality or their potential impact. Therefore, our objective was to analyze the presence of biases in articles, evaluate the use of AI models for cancer diagnosis, and assess the quality of these articles. Additionally, we evaluated the potential impact of these studies by examining the citation counts and determining whether the evaluated models have been utilized in subsequent research. Our study focused on JCO Clinical Cancer Informatics, which is recognized as one of the leading journals in bioinformatics and artificial intelligence research applied to cancer. Additionally, this journal is affiliated with the ASCO, one of the most prestigious scientific societies in oncology research, and is known for establishing the six core principles for the responsible use of artificial intelligence in oncology.

## 2. Materials and Methods

### 2.1. Eligibility Criteria

The articles must represent original research concerning the application of AI techniques for cancer diagnosis. This study exclusively evaluated predictive AI models for the screening or diagnosis of various types of cancer, excluding AI models aimed at other outcomes such as survival, prognosis, staging, or treatment. We focused on cancer diagnosis because the consequences of missed cancer diagnosis or mistaken cancer diagnosis can result in significant harm to patient health and well-being [12]. The delay in cancer diagnosis results in patients’ cancer being diagnosed at later stages, which limits the treatment options and results in shortened survival [13]. The incorrect diagnosis of cancer results in overtreatment and a reduced quality of life [14]. All eligible articles published in the JCO Clinical Cancer Informatics journal were included in the analysis since its first issue. The journal was chosen as a highly regarded source of publications specializing in data science and predictive modeling in cancer and includes a broad spectrum of cancer-related topics. Although it is an official ASCO journal, the extent of its adherence to the ASCO recommendations on responsible AI applications in oncology is unknown. Articles with participants of any age group, sex, race, ethnicity, or other sociodemographic or clinical characteristics are eligible. Articles from both observational and experimental study designs are included. Reviews and other secondary research articles are excluded.

### 2.2. Search Strategy and Sources

A search query incorporating terms related to oncology and AI was developed and filtered via the journal field to include only articles published in the target journal (Box 1). The query was implemented in PubMed (MEDLINE) from its inception to 29 July 2024.

Box 1 **Search query.**

((“Neoplasms”[Mesh] OR cancer[tiab] OR neoplasm*[tiab] OR oncolog*[tiab]) AND (“Artificial Intelligence”[Mesh] OR artificial intelligence[tiab] OR deep learning[tiab] OR machine learning[tiab] OR supervised learning[tiab] OR unsupervised learning[tiab] OR reinforcement learning[tiab])) AND (“JCO clinical cancer informatics”[Journal])

### 2.3. Screening and Data Extraction

We utilized the Rayyan web platform [15] for the selection process. Two reviewers (CMRR and SEA) initially reviewed the titles and abstracts of the articles resulting from the search query. The screened articles were then reviewed in full text, independently, and duplicated by the reviewers. A third reviewer (DVZ) made the final selection decision when disagreements arose.

We developed a collection form in MS Excel for the data extraction process. Each reviewer independently collected information using the form. The inclusion form comprised information regarding the first author’s name, year of publication, article title, patient characteristics, type of AI tested or developed, clinical settings, reported outcomes (AI performance metrics), citation according to the Google Scholar search engine, and quality assessment via the CREMLS checklist [16]. Two reviewers (DVZ and SEA) also independently performed data extraction.

### 2.4. Outcomes

#### 2.4.1. Characteristics of the Included Studies

We describe the participants’ characteristics based on each of the AI analysis’s phases (i.e., training, testing, and validation). We present information on cancer type, age groups, sex, and other sociodemographic variables.

#### 2.4.2. AI Performance Metrics

Our study reported the primary performance metrics for each AI model evaluated in the included articles. The metrics analyzed were sensitivity (recall or true positive rate), specificity (true negative rate), accuracy (probability of correct classification), precision (positive predictive value), F1 score, and ROC/AUC. We report the metrics for each model evaluated and indicate which model was the best-performing model highlighted in each study.

#### 2.4.3. AI Biases

To evaluate the potential biases within the AI models across the included studies, we compiled a set of criteria and designed a detailed assessment tool for each (see Supplementary Material Table S1). This tool is based on three principal sources that guide identifying and evaluating the biases likely to impact the AI models used for cancer prediction.

First, we established minimum AI bias criteria for cancer by following the “Principles for Responsible Use of AI in Oncology” [10]. These principles provide guidelines on the study, implementation, and ethical use of AI in oncology. The criteria include the following: (1) transparency throughout the entire AI life cycle, (2) stakeholder awareness of AI usage, (3) fairness and impartiality, (4) accountability and compliance with local regulations, (5) oversight and privacy, and (6) human-centered AI application.

Second, we incorporated the potential sources of bias to which AI models in oncology may be susceptible, as identified by Dankwa-Mullan and Weeraratne [8]. These authors reviewed the potential biases, disparities, and diversity issues in AI models for cancer, identifying five additional sources of potential bias: (7) research bias, (8) provider expertise bias, (9) embedded data bias, (10) environmental and life-course bias, and (11) empathy or contextual bias.

Finally, we included additional sources of bias and mitigation strategies in AI models, applicable to both cancer and other health concerns, as proposed by Chen et al. [9]. These authors identified six previously unaddressed sources of bias risk: (12) implicit bias, (13) selection bias, (14) measurement bias, (15) confounding bias, (16) algorithmic bias, and (17) temporal bias.

Therefore, our evaluation tool encompassed seventeen criteria, each aimed at identifying a specific source of bias that could compromise the accuracy, fairness, and applicability of AI models for cancer prediction.

Additionally, our assessment of potential biases included a comprehensive evaluation of the potential risks of bias based on the participants, outcomes, analysis, and predictors. For this purpose, we utilized the PROBAST tool (Prediction model Risk of Bias Assessment Tool, https://www.probast.org/, accessed on 22 January 2025) [17].

### 2.5. Quality Assessment

Three authors were trained in interpreting and using the “Consolidated Reporting Guidelines for Prognostic and Diagnostic Machine Learning Modeling Studies” (CREMLS) checklist [16]. The CREMLS checklist comprises 35 items across five sections (study details, data, methodology, evaluation, and explainability and transparency). Each item was marked as present (yes), absent (no), unclear, or not applicable. We conducted pilot testing for article screening, data extraction, and quality evaluation processes with 10% of the articles. We evaluated and reported decision concordance using the Kappa statistic, considering a Kappa value greater than 0.8, indicating high consistency.

The CREMLS checklist is a specific tool designed to assess the quality of reporting in primary studies on diagnostic models utilizing AI. It includes a comprehensive set of items that evaluate the technical reporting of AI models, distinguishing it from other tools such as transparent reporting of a multivariable prediction model for individual prognosis or diagnosis (TRIPOD) [18]. Although a specific version for AI models, TRIPOD-AI [19], is now available, we consider this checklist to primarily serve as a guide for reporting rather than providing an in-depth technical evaluation, as is achieved with the CREMLS checklist.

### 2.6. Potential Impact

The potential impact was assessed based on the total citation count and average citations per year. Additionally, we examined the citations to include studies to evaluate whether these studies or their AI models were replicated or used in the subsequent research. This approach provides an insight into whether the studies have been applied, replicated, or implemented in contexts beyond the original research.

We used Google Scholar for citation tracking, as it includes a broad range of sources—peer-reviewed articles, preprints, theses, technical reports, and other documents—offering greater sensitivity for our study’s purposes. A sub-analysis evaluated the association between AI performance metrics and citation counts, that is, to evaluate whether studies with higher AI performance metrics received more citations compared to those with lower metrics. A multiple linear regression model, adjusted for the publication year, was used, with the citation count as the outcome and AI performance metrics as exposures.

### 2.7. Statistical Analysis

All statistical analyses were performed using R 4.4.2 version [20]. Categorical data were reported using percentages and absolute frequencies. 

## 3. Results

### 3.1. Study Selection

**Figure 1:**
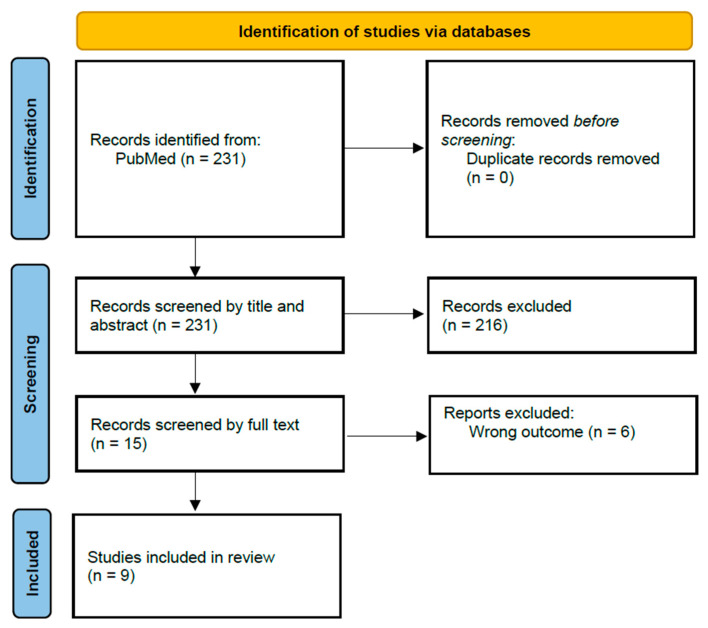
Flowchart diagram illustrating the study selection process for the systematic review, following the PRISMA 2020 guidelines [30].

Our search identified 231 records in PubMed, which were screened by title and abstract, excluding 216 records. A total of 15 records underwent a full-text review, of which six were excluded. The main exclusion criteria during the full-text review were as follows: studies focusing on prognosis rather than diagnosis (*n* = 2), studies centered on improving the image quality rather than performing diagnosis or screening (*n* = 1), studies addressing AI applications for prognosis and classification (*n* = 1), studies that focused on extracting data from electronic health records (EHR) (*n* = 1), studies assessing the stability of AI (*n* = 1), and studies describing the existing tools without developing or validating an AI model (*n* = 1). The reasons for exclusion are detailed in Supplementary Material Table S2. Therefore, nine articles were included in our study [21,22,23,24,25,26,27,28,29] (see Figure 1).

### 3.2. Characteristics of the Included Studies

Our study identified seven studies that reported descriptive information about the included participants [22,23,25,26,27,28,29], and two studies that reported descriptive information about the features or images used in their analysis but did not provide information on the number of participants in the training, testing, or validation phases [21,24]. Most studies were conducted on U.S. populations (6/9, 66%), while the remainder were conducted in Denmark, Germany, and Japan. Most of the studies were conducted in a general hospital setting (3/9, 33%), in Veterans Affairs Medical Centers (2/9, 22%), and in primary care and hospital facilities nationwide (2/9, 22%). All studies reported a training phase, five reported a testing phase [22,23,25,27,28], and four reported a validation phase [23,24,25,28]. The majority of the studies focused on pancreatic cancer (4/9, 44%), while the remaining studies evaluated other cancers such as bladder cancer, hepatocellular carcinoma, ovarian cancer, small-cell lung cancer, and urothelial carcinoma. Four studies focused on cancer diagnosis [23,26,27,28], and five focused on early detection, identification, or cancer screening [21,22,24,25,29]. The specific characteristics of each study can be found in Table 1. A detailed description of the characteristics of the studies included, by phase, is presented in Supplementary Material.

### 3.3. AI Performance Metrics

We identified a total of 24 different AI models used across the studies, with an average of 3.6 models evaluated per study (range: 1 to 7 models). The most commonly used AI models were Random Forest (5/9) [21,25,26,27,29], Support Vector Machine (SVM) (2/9) [25,27], Logistic Regression (2/9) [27,29], Gradient Boosting [25,27], XGBoost [22,29], and the Ensemble Model (2/9) [25,27]. Regarding model performance, the models that showed the best performance were primarily the Random Forest models (3/9) [21,26,29] and the Ensemble Model (2/9) [25,27]. It is noteworthy that in studies where Ensemble Models were used, these were reported as the best-performing models. However, their composition varied greatly between the studies and depended on the models previously evaluated. The metrics for each study are presented in Table 2.

Most studies reported the performance metrics from the testing phases (5/9) [21,25,26,27,28], three studies reported the performance metrics from the validation phases [24,25,28], and two studies reported the performance metrics from the training phases [25,29]. One study reported the performance metrics from a combination of the training and testing phases [22]. In one study, it is unclear at what stage the performance metrics were reported [23]. Notably, only one study reported the performance metrics for all three phases: training, testing, and validation [25].

Regarding the performance metrics, most of the studies reported sensitivity (7/9), ROC/AUC (7/9), specificity (6/9), and precision (6/9). The studies evaluated different types of cancer using different models and populations, making direct comparisons between them difficult. However, among the four studies that evaluated pancreatic cancer (4/9), the study by Firpo and collaborators showed the highest values for sensitivity, specificity, accuracy, and ROC/AUC [25]. Despite this, the study did not report precision and F1 scores.

It should be noted that most of the studies reported high values for the different AI performance metrics evaluated, with values ranging from 1.00 to 0.80 (see Supplementary Material Table S3).

### 3.4. AI Biases

**Figure 2:**
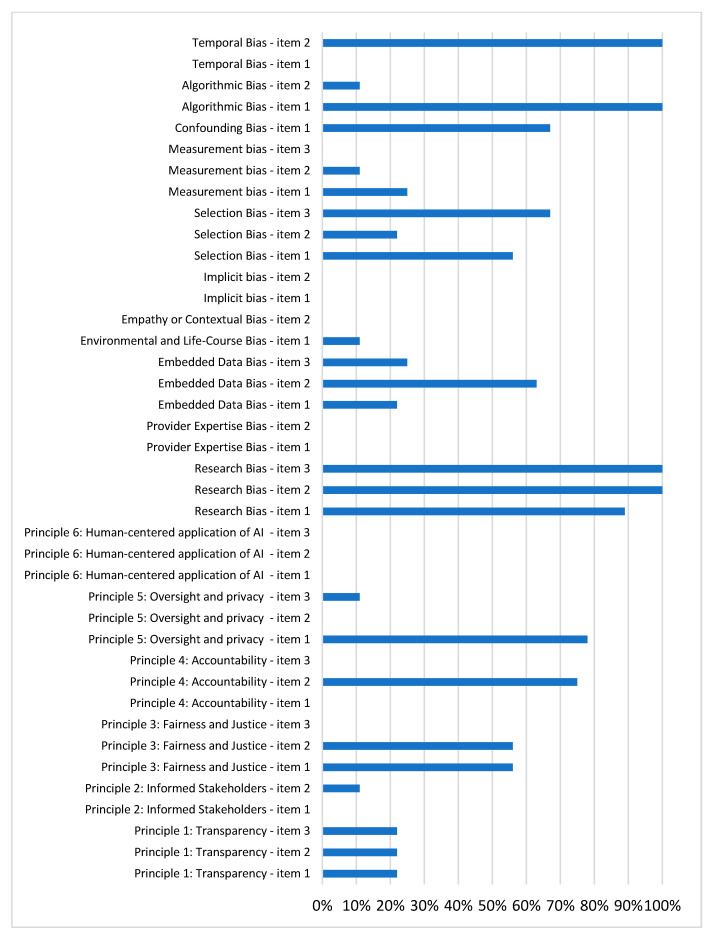
Distributional biases of reviewed articles.

Our study evaluated ten potential biases in AI models and presents the distribution of compliance with the reporting practices aimed at mitigating these biases in Figure 2. Higher percentages indicate that the studies either controlled for the potential source of bias or provided information that would facilitate its control. A detailed explanation of each individual bias is provided below.

#### 3.4.1. Principle 1: Transparency

We evaluated whether the included articles complied with the first three principles for the responsible use of artificial intelligence in oncology of the American Society of Clinical Oncology. When analyzing the compliance with the first principle of transparency, we found that while all articles explained how their models were designed and presented one or more metrics to evaluate the models, only 22% of the studies (2/9) shared both their training and test datasets, and provided access to study reproducibility materials such as code, hyperparameters, and procedures [24,25]. The different criteria used to evaluate the AI bias are presented in Table 3.

#### 3.4.2. Principle 2: Informed Stakeholders

Regarding the second principle, Informed Stakeholders, none of the included studies reported whether healthcare professionals or users had been trained to interpret the AI model’s results. Additionally, only one study explicitly mentioned that its data would be used to train an AI model (1/9, 11%) [24]. 

#### 3.4.3. Principle 3: Fairness and Justice

We assessed the adherence to the principles of justice and equity within the articles. Regarding the use of metrics to evaluate the model fairness, 56% of the studies (five out of nine) met this criterion, providing specific metrics for subgroups based on age, gender, cancer type, and specific comorbidities [21,22,24,28,29]. 

Additionally, for the criterion concerning participant diversity, 56% (five out of nine) of the studies reported sociodemographic information for the participants across the model’s training, testing, and validation phases [25,26,27,28,29]. However, none of the studies reported using specific ethical guidelines for AI models to ensure fairness and justify outcomes.

#### 3.4.4. Principle 4: Accountability and Compliance with Local Regulations

Only 75% (6/8) of the studies indicated that they had obtained approval from an ethics committee or had undergone an ethical review extension [25,26,27,28,29].

#### 3.4.5. Principle 5: Oversight and Privacy

Seventy-eight percent (7/9) of the included studies detailed the measures taken to protect participant privacy and confidentiality, such as the use of anonymization techniques [21,23,24,25,26,27,29]. However, only one study discussed the role of ensuring the autonomy of healthcare professionals and patients in the context of using AI models for cancer diagnosis [23]. Additionally, none of the studies reported the use of privacy-enhancing technologies to ensure that the data sharing was conducted privately and confidentially.

#### 3.4.6. Principle 6: Human-Centered AI Application

Our findings indicate that none of the studies reported the intentions of human involvement in AI-assisted healthcare decision making. Specifically, they did not state that the AI model does not replace human interaction, ensure human oversight throughout the AI model lifecycle, or confirm verification and final approval of healthcare professionals’ consent before making any clinical decisions.

#### 3.4.7. Research Bias

In analyzing the research bias, 89% of the studies (8/9) provided evidence of incorporating real-world data to enhance the model findings [21,22,23,24,25,26,27,28]. Also, all the studies reported the research team as having a diverse background. Furthermore, all the studies reported the disclosed funding sources, potential conflicts of interest, or policy decisions that may have influenced the research. Notably, some of the studies reported funding from private companies or the pharmaceutical industry, suggesting potential conflicts of interest warranting consideration.

#### 3.4.8. Provider Expertise Bias

None of the studies considered the potential biases, inconsistencies, or stereotypes from healthcare providers that might impact the data or influence the application of the AI model. Additionally, none of the studies reported that consistent clinical guidelines were used for care and data collection across all the participants.

#### 3.4.9. Embedded Data Bias

Twenty-two percent of the studies (2/9) analyzed the potential biases in the data or data collection processes [24,26]; however, in two cases, it was unclear whether this criterion was fully met or only partially addressed [21,27]. Additionally, 63% (5/8) of the studies incorporated synthetic or imputed data for model training [23,24,27,28,29], yet only 25% (2/8) discussed handling missing or incomplete data to avoid introducing bias [27,28]. Notably, one study reported having all the data, making this criterion inapplicable [25].

#### 3.4.10. Environmental and Life-Course Bias

Only one study (11%) assessed whether environmental, occupational, or lifetime factors may have influenced the AI model’s results [26]. The remaining studies did not assess this criterion.

#### 3.4.11. Empathy or Contextual Bias

None of the studies reported information on the authors’ knowledge or experience related to the people, culture, or contextual factors within the data used for the AI model. This gap raises the possibility that contextual factors may not have been fully considered during data coding, analysis, or the publication process.

#### 3.4.12. Implicit Bias

None of the studies reported or discussed the presence of potential preexisting biases, such as stereotypes or flawed assumptions, in the data used. Additionally, they did not address whether implicit bias is associated with negative clinical interactions and poorer treatment outcomes for vulnerable patients.

#### 3.4.13. Selection Bias

Fifty-six percent of the studies (5/9) evaluated whether the data used to train the AI model was representative of the target population [21,22,23,27,29]. However, this criterion needed to be clarified in the remaining studies. Only two studies (22%) also trained their models on datasets representative of diverse demographic groups and health conditions [22,27]. Moreover, 67% (6/9) analyzed or discussed whether the sampling methods may have introduced bias, potentially leading to the underrepresentation or overrepresentation of certain groups [21,24,25,26,27,28].

#### 3.4.14. Measurement Bias

Of the included studies, 25% (2/8) evaluated the presence of inaccuracies or incomplete data entries by clinicians or clinical devices during the data collection process [22,27]. Only 11% (1/9) discussed the measures taken to mitigate the measurement bias and ensure consistency among the participants, such as using guidelines or equipment calibration [27]. Notably, none of the studies (0%) assessed how the inaccuracies in the clinical device data might impact the outcomes of the AI model.

#### 3.4.15. Confounding Bias

Regarding confounding bias, 67% of the studies (6/9) assessed the possibility of confounding factors that could distort the relationship between the input variables and the results [21,22,25,26,27,28]

#### 3.4.16. Algorithmic Bias

All the included studies evaluated the performance metrics of the AI models for at least one metric. However, not all the models presented all the possible metrics, which could suggest the selective reporting of the performance metrics. It should be noted that only one study reported information on the evaluation or fulfillment of the assumptions supporting the use of the statistical analyses performed [21].

#### 3.4.17. Temporal Bias

Regarding temporal bias, none of the studies evaluated or discussed the possible contextual changes that might have influenced the data collection, model training, or its predictive ability over time. However, it is important to note that only one study addressed the analysis of temporally nested data, such as video frames [24].

#### 3.4.18. Risk of Bias Using PROBAST

The PROBAST analysis revealed that 67% (6/9) of the studies exhibited a low overall risk of bias, and 78% (7/9) demonstrated a low risk of bias for the potential applications of the models in subsequent real-world studies. The evaluation of the different PROBAST criteria is provided in Supplementary Material Table S4.

### 3.5. Quality Assessment

**Figure 3:**
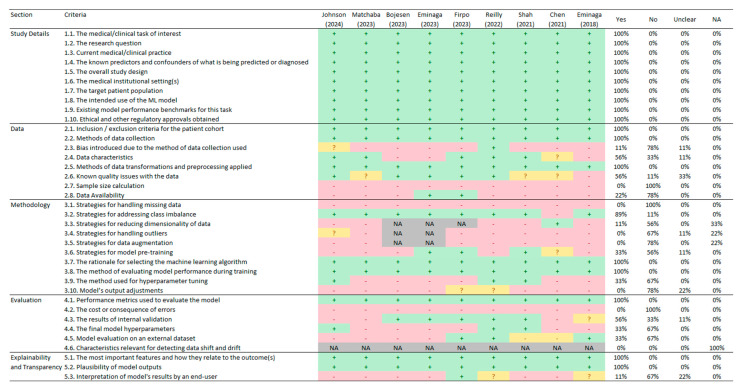
Consolidated reporting guidelines for prognostic and diagnostic machine learning model check-list (*n* = 9) [21,22,23,24,25,26,27,28,29]. Note: NA = not applicable (in grey). + = Yes (in green). - = No (in red). ? = unclear (in yellow).

Upon applying the CREMLS checklist, we identified that all the studies met the ten criteria in the first section of the study details, which cover the basic aspects of the study, such as the research question, study design, participant description, and the intention to implement an AI model (see Figure 3). 

In the second section of the data, a lower proportion of the criteria were fulfilled. While the criteria related to the inclusion and exclusion criteria (criterion 2.1), data collection methods (criterion 2.2), and data transformations and preprocessing (criterion 2.5) were reported in all the studies, only two studies shared their data (criterion 2.8) [24,25], and only one study explicitly reported the potential biases introduced by the data collection method used (criterion 2.3) [28]. Moreover, none of the studies calculated a sample size to estimate whether they had sufficient participants or images for the necessary analyses (criterion 2.7).

In the analysis of the third section of the methodology, a lower proportion of compliance was identified in several key areas. None of the studies addressed the strategies for handling missing data (criterion 3.1), managing outliers (criterion 3.4), data augmentation strategies (criterion 3.5), or adjustments to the model outputs (criterion 3.10). Additionally, only 11% of the studies implemented strategies for dimensionality reduction (criterion 3.3) [22], 33% reported on model pretraining (criterion 3.6), and 33% detailed the method used for hyperparameter tuning (criterion 3.9).

Regarding the fourth section of the evaluation, all the studies reported at least one metric to assess the model (criterion 4.1). However, none of the studies reported the costs or consequences of errors (criterion 4.2) or characteristics relevant for detecting data shift and drift (criterion 4.6). Also, 33% of the studies reported the final model hyperparameters (criterion 4.4) and the evaluation of the model on an external dataset (criterion 4.5).

Finally, in the fifth section of explainability and transparency, we found that all the studies reported the most important features and how they related to the outcome (criterion 5.1) as well as the plausibility of the model’s outputs (criterion 5.2). However, only one study reported the interpretation of the model’s results by an end-user (criterion 5.3) [25].

### 3.6. Potential Impact

We found that the included studies were cited a total of 128 times (mean = 14.2, range = 0 to 71), with the most frequently cited one being the paper by Eminaga [23]. However, only two studies have been replicated by other research teams. On one hand, the AI model proposed by Reilly [28] was used in three subsequent studies [31,32,33]. On the other hand, the model by Eminaga [23] was utilized in only one follow-up study [34]. The average number of citations per year and the total number of citations per article are presented in Table 4.

Additionally, our sub-analysis of the association between the AI performance metrics and the number of citations found no correlation between the reported metric values and the number of citations received by the study (see Supplementary Material Table S5).

## 4. Discussion

Our study identified the potential biases in the included studies evaluating AI models for cancer diagnosis. None of the studies reported information related to empathy or contextual bias, implicit bias, provider expertise bias, or the sixth ASCO principle on the human-centered application of AI in oncology. Additionally, many studies exhibited potential biases associated with environmental and life-course bias, measurement bias, and embedded data bias, as they did not provide details on the data collection processes, standards applied for data collection, or the management of missing or incomplete data. Furthermore, the compliance with the remaining five ASCO principles for responsible AI use in oncology was very limited. Consequently, our analysis found that various sources of bias may influence the reported outcomes in the included studies. It is important to note that the low compliance rate with the ASCO’s last three principles for AI in cancer (principles 4, 5, and 6) is understandable, as these criteria are primarily relevant to the models implemented in real-world settings [10], while the studies included in our review were conducted in controlled research environments.

In addition, we identified that most of the studies did not adhere to the guidelines for machine learning models in prognosis and diagnosis (CREMLS checklist), particularly in the sections concerning data, methodology, and evaluation. The reporting of these investigations is often limited, especially in terms of the AI performance metrics, participant description, and methodology reporting. It is worth noting that both the first ASCO principle on transparency and the CREMLS checklist criterion highlight the need for data sharing. However, only 22% of the studies (two out of nine) provided information on the data and procedures necessary for study replicability. Therefore, cancer screening and diagnostic studies using AI need to improve the quality of their reporting and increase the transparency by making the data available to enhance the reproducibility of the studies [35].

Our evaluation of the potential impact of the publications identified that 22% of the included studies (two out of nine) have been replicated or utilized by other research teams, all of whom obtained results that support the original findings and the use of AI models for cancer diagnosis [31,32,33,34]. This suggests that while the results are promising, the proportion of replicated studies remains limited. It is noteworthy that most of the included studies were published between 2023 and 2024, which may not yet have allowed sufficient time to fully assess their impact.

At the level of the reported AI performance metrics, our study found that most of the reviewed articles applying AI models in oncology reported the performance metrics in the high to moderate range for sensitivity, specificity, accuracy, and ROC/AUC. This could potentially obscure null or negative results and introduce a potential bias in favor of publishing positive outcomes [36]. However, no formal publication bias test was conducted due to the nature of the studies and their lack of direct comparability. Also, previous systematic reviews have found no evidence of publication bias when evaluating the use of artificial intelligence for detecting gastric precancerous lesions, skin cancer, or adenomas and polyps [37,38,39]. Furthermore, the reporting of the AI performance metrics varied greatly among the different studies, so it is necessary to standardize the reporting of the different metrics.

The high prevalence of cancer within the studies included suggests that AI-based diagnostic tools should primarily be implemented in hospital settings, where the likelihood of encountering cancer cases is higher [40]. In contrast, deploying these tools in community or primary care settings may be less effective due to the lower prevalence of cancer in these contexts and the fact that the AI models were not trained under such conditions[40]. This underscores the importance of carefully considering the context in which AI technologies are implemented to ensure accurate diagnoses and improved patient outcomes. Although some AI-based products have received approval from the U.S. Food and Drug Administration (FDA), their integration into real-world clinical workflows remains limited [41]. Nonetheless, the incorporation of AI models for cancer detection in clinical practice shows the potential for increasing the number of detected cases and partially alleviating the workload of healthcare professionals [42]. However, it is crucial to recognize that these outcomes may vary depending on the specific AI model utilized and the characteristics of the patient population included in the training datasets.

Our study also found that most of the reviewed studies involved small datasets, posing challenges for developing AI models that adequately represent diverse sociodemographic characteristics and ensure equitable predictive performance. One potential solution is to utilize privacy-enhancing technologies to ensure that data sharing is conducted privately and confidentially. However, data protection laws in the healthcare field often limit or complicate the sharing of large volumes of medical information. Therefore, ethical and legal standards must be followed to regulate the sharing of medical data for research purposes [43,44]. Another alternative is generating synthetic data through machine learning techniques, such as generative adversarial networks, which can simulate underrepresented populations and balance the demographic characteristics within datasets [45,46].

### Strengths and Limitations

Our study has some limitations. First, we included only one journal in our review, which limits the generalizability of the findings to the broader body of scientific evidence. Second, many of the studies lacked detailed participant characteristics and AI performance metrics during the training, testing, and validation phases, making it challenging to evaluate the applicability of the models to diverse populations. Notably, the absence of precise sociodemographic data could result in the underrepresentation of certain groups, potentially leading to biased diagnostic decisions that disproportionately impact vulnerable populations. Addressing this issue will require future research to comprehensively and transparently report the participant characteristics. Third, all of the included studies were from high-income countries, so the results may not be applicable to low- and middle-income countries.

The main strength of our study lies in its comprehensive evaluation of the potential sources of bias in studies assessing AI models for cancer prediction, grounded in prior research and the principles for the responsible use of AI in oncology of the ASCO [8,9,10]. Additionally, we assessed the quality of these studies from multiple perspectives, including the use of the CREMLS checklist and potential impact metrics. This multifaceted approach enabled us to identify the significant deficiencies in transparency and reporting quality across the reviewed studies.

## 5. Conclusions

Our study reveals that most of the reviewed articles on the use of artificial intelligence in cancer diagnosis and screening presented potential risks of bias, demonstrated partial adherence to the ASCO’s principles for responsible AI use in oncology, and showed deficiencies in the reporting quality, particularly regarding data, methodology, and evaluation. Therefore, the authors recommend promoting greater transparency, data accessibility, and adherence to the ASCO’s established guidelines, thereby improving the reproducibility and reliability of study results.

## Figures and Tables

**Table 1:** Characteristics of the included studies (*n* = 9).

Author (year)	Country	Type of Cancer	Objective	Training	Testing	Validation	Clinical Setting	Funding
Johnson (2024) [26]	Japan	Hepatocellular carcinoma	Diagnostic	Overall: 3473 patients (49.4% male, median age 61.0 years). With cancer: 445 (12.8%) patients (60.4% males, median age 65.0 years). Without cancer: 3028 (87.2%) patients (47.8% male, median age 60.3 years).	Not reported	Not reported	Single general hospital	Private company
Matchaba (2023) [27]	United States	Pancreatic cancer	Diagnostic	Overall: 15,189 participants (35% male, median age 60 years). With cancer: 8438 (56%) patients (51% male, median age 67 years). Without cancer: 6751 (44%) patients (15% male, median age 52 years).	Overall: 3798 participants (35% male, median age 60 years). With cancer: 2127 (56%) patients (51% male, median age 67 years). Without cancer: 1671 (44%) patients (15% male, median age 53 years).	Not reported	Primary care and hospital facilities nationwide	Pharmaceutical industry
Bojesen (2023) [21]	Denmark	Pancreatic cancer	Screening	Overall: Case-control dataset. Median age: 71 years (case and control groups). Gender not specified.	Not reported	Not reported	Primary care and hospital facilities nationwide	Foundation
Eminaga (2023) [24]	United States	Bladder cancer	Screening	Overall: 312 images used (number of patients not specified). Gender and age not reported.	Not reported	Overall: Videos from 68 cases with 272,799 frames. With cancer: 84,579 (31.0%) frames were labeled as regions with cancer. Without cancer: 188,220 (69.0%) frames were labeled as regions without cancer. Gender and age not reported.	Veterans Affairs Medical Centers	Private company
Firpo (2023) [25]	United States	Pancreatic cancer	Screening	Overall: 669 patients (60% male, median age 59 years). With cancer: 152 (19%) patients (57% male, median age 67 years). Without cancer: 517 (81%) patients (56% male, median age 57 years).	Overall: 168 participants (47% male, median age 62 years). With cancer: 30 (18%) patients (60% male, median age 69 years). Without cancer: 138 (82%) patients (44% male, median age 60 years).	Overall: 186 participants (47% male, median age 62 years). With cancer: 73 (39%) patients (47% male, median age 69 years). Without cancer: 113 (61%) patients (46% male, median age 58 years).	Single specialized hospital (cancer)	Government entity
Reilly (2022) [28]	United States	Ovarian Cancer	Diagnostic	Overall: 853 patients (all are women, median age 51.3 years). With cancer: 280 (33%) patients (median age not reported). Without cancer: 573 (67%) patients (median age not reported).	Overall: 214 patients (all are women, median age 50.8 years). With cancer: 56 (26%) patients (median age not reported). Without cancer: 158 (74%) patients (median age not reported).	Overall: 2000 patients (all are women, median age 47.5 years). With cancer: 98 (4.9%) patients (median age not reported). Without cancer: 1902 (95.1%) patients (median age not reported).	Unclear	Private company
Shah (2021) [29]	United States	Small-Cell Lung Cancer	Screening	Overall: 103 patients (98% male, median age 73 years). With cancer: 26 (25%) patients (100% male, median age 75.6 years). Without cancer: 77 (75%) patients (97% male, median age 72.1 years).	Not reported	Not reported	Veterans Affairs Medical Centers	Government entity and University
Chen (2021) [22]	United States	Pancreatic cancer	Screening	Overall: 56,474 patients (41% male, median age 59 years). With cancer: 3322 (5.88%) patients (50.5% male, median age 66.5 years). Without cancer: 53,152 (94.12%) patients (40.7 male, median age 59 years).	30% of the total dataset. No information on age, sex, or by patients with and without cancer is reported.	Not reported	Single general hospital	Government entity
Eminaga (2018) [23]	Germany	Urothelial carcinoma	Diagnostic	Overall: 479 patients, encompassing 44 urological findings. No information on age, sex, or by patients with and without cancer is reported.	30% of the total dataset. No information on age, sex, or by patients with and without cancer is reported.	10% of the total dataset. No information on age, sex, or by patients with and without cancer is reported.	Single general hospital	Foundation

**Table 2:** AI performance metrics (*n* = 9).

Author (year)	Stage	Models Evaluated	Sensitivity (Recall or True Positive Rate)	Specificity (True Negative Rate)	Accuracy (Probability of Correct Classification)	Precision (Positive Predictive Value)	F1	ROC/AUC
Johnson (2024) [26]	Testing	RF-GALAD: Based on variables from the GALAD model (best model) RF-practical: Based on routine clinical and serological biomarkers (best model)	**RF-GALAD: 90.7%** **RF-practical: 85.9%**	**RF-GALAD: 74.5%** **RF-practical: 86.7%**	**RF-GALAD: 82.6%** **RF-practical: 82.0%**	**RF-GALAD: 0.477** **RF-practical: 0.467**	**RF-GALAD: 0.623** **RF-practical: 0.614**	**RF-GALAD: 0.907** **RF-practical: 0.911**
Matchaba (2023) [27]	Testing	SVM, RF, DT, LR, GB, EM (merges SVM, RF, DT, LR, and GB) (best model)	SVM: 41.84% RF: 95.58% DT: 72.87% LR: 93.27% GB: 84.91% **EM: 85.61%**	SVM: 71.39% RF: 38.42% DT: 69.71% LR: 13.22% GB: 75.88% **EM: 76.18%**	Not reported	Not reported	SVM: 0.5093 RF: 0.7835 DT: 0.7410 LR: 0.7135 GB: 0.8330 **EM: 0.8380**	SVM: 0.53 RF: 0.80 DT: 0.71 LR: 0.61 GB: 0.88 **EM: 0.89**
Bojesen (2023) [21]	Testing	RF (best model), BT	23.4% (in Combined cohorts) Data reported only from the best model	Not reported	Not reported	10.1% (in Combined cohorts) Data reported only from the best model	Not reported	74.4% (in Combined cohorts) Data reported only from the best model
Eminaga (2023) [24]	Validation	ConvNeXt (best model), PlexusNet (best model), MobileNet, SwinTransformer.	Frame level: 81.4% to 88.1% Block level: 100% Does not report the value per model.	Frame level: 30.0% to 44.8% Block level: 56% to 67% Does not report the value per model.	Not reported	32.8% to 37.0% Does not report the value per model.	0.444 to 0.495 Does not report the value per model.	63.9% to 74.4% Does not report the value per model.
Firpo (2023) [25]	Training, Testing, and Validation	GLMnet, RF, KNN, SVM, NNET, EM using stacking (merges GLMnet, KNN, NNET, RF, and SVM) (best model)	Training set: 92.8% Test set: 63.3% Validation set: 72.6% Data reported only from the best model	Training set: 99.8% Test set: 97.1% Validation set: 95.6% Data reported only from the best model	Training set: 98.2% Test set: 90.5% Validation set: 86.6% Data reported only from the best model	Not reported	Not reported	Training set: Not reported Test set: 0.944 Validation set: 0.925 Data reported only from the best model
Reilly (2022) [28]	Testing and Validation	MIA3G	Test Dataset Overall: 91.07% Premenopausal: 88.89% Postmenopausal: 92.11% Epithelial ovarian cancer: 93.33% Validation Dataset: Overall: 89.80% Premenopausal: 80.77% Postmenopausal: 93.06% Epithelial ovarian cancer: 94.94%	Test Dataset Overall: 87.97% Premenopausal: 95.40% Postmenopausal: 78.87% Validation Dataset: Overall: 84.02% Premenopausal: 91.86% Postmenopausal: 71.56%	Not reported	Testing Dataset Overall: 72.86% Premenopausal: 80.00% Postmenopausal: 70.00% Validation Dataset: Overall: 22.45% Premenopausal: 18.10% Postmenopausal: 24.28%	Not reported	Test Dataset: Overall: 0.938 Validation Dataset: Overall: 0.937
Shah (2021) [29]	Training	LR, RF (best model), SVC, XGBoost	Not reported	Not reported	Not reported	Not reported	Not reported	Noncontrast scans: **RF: 0.81** SVC: 0.77 XGBoost: 0.84 LR: 0.84. Contrast-enhanced scans: **RF: 0.88** SVC: 0.87 XGBoost: 0.85 LR: 0.81
Chen (2021) [22]	Merge between training and testing	XGBoost (best model)	60%	90%	Not reported	0.07% to 0.23%	Not reported	0.84
Eminaga (2018) [23]	Unclear	ResNet50, VGG-19, VGG-16, InceptionV3, Xception (best model), harmonic-series concept, 90%-layer concept	Not reported	Not reported	**Xception: 99.52%** ResNet50: 99.48% InceptionV3: 98.73% Harmonic-series concept: 99.45% 90%-layer concept: 99.11% VGG-16: 97.42% VGG-19: 95.47%	**Xception: 99.54%** ResNet50: 99.48% InceptionV3: 98.86% Harmonic-series concept: 99.45% 90%-layer concept: 99.11% VGG-16: 97.82% VGG-19: 95.65%	**Xception: 0.9952** ResNet50: 0.9948 InceptionV3: 0.9874 Harmonic-series concept: 0.9945 90%-layer concept: 0.9911 VGG-16: 0.9735 VGG-19: 0.9547	Not reported

Note: RF = Random Forest. SVC = Support Vector Classifier. SVM = Support Vector Machine. DT = Decision Tree. BT = Boosted Trees. LR = Logistic Regression. GB = Gradient Boosting. EM = Ensemble Model. GLMnet = elastic-net regularized generalized linear model. KNN = k-nearest neighbors. NNET = neural networks. Bolded values refer to the best model, when multiple models were evaluated.

**Table 3:** Potential biases in artificial intelligence models for predicting cancer (*n* = 9).

Bias Criterion	Items	[26]	[27]	[21]	[24]	[25]	[28]	[29]	[22]	[23]	Yes % (n)
Principle 1: Transparency	1. Data and procedure access of the training	No	No	No	Yes	Yes	No	No	No	No	22% (2/9)
	2. Data and procedure access of the testing	No	No	No	Yes	Yes	No	No	No	No	22% (2/9)
	3. Reproducibility materials access	No	No	No	Yes	Yes	No	No	No	No	22% (2/9)
Principle 2: Informed Stakeholders	1. Professional training in AI usage	No	No	No	No	No	No	No	No	No	0% (0/9)
	2. Patient’s informed consent for the use of data in AI.	No	No	No	Yes	No	No	No	No	No	11% (1/9)
Principle 3: Fairness and Justice	1. Model fairness measures	No	No	Yes	Yes	No	Yes	Yes	Yes	No	56% (5/9)
	2. Diversity of participants included is reported	Yes	Yes	No	No	Yes	Yes	Yes	No	No	56% (5/9)
	3. Compliance with specific ethical guidelines for AI models	No	No	No	No	No	No	No	No	No	0% (0/9)
Principle 4: Accountability	1. Compliance with legal and regulatory requirements	No	No	No	No	No	No	No	No	No	0% (0/9)
	2. Adherence to ethical standards	Yes	NA	Yes	Yes	Yes	Yes	Yes	No	No	75% (6/8)
	3. Statement of responsibility	No	No	No	No	No	No	No	No	No	0% (0/9)
Principle 5: Oversight and privacy	1. Patient data privacy protection	Yes	Yes	Yes	Yes	Yes	No	Yes	No	Yes	78% (7/9)
	2. Use of privacy-enhancing technologies	No	No	No	No	No	No	No	No	No	0% (0/9)
	3. Ensuring the autonomy of health professionals and patients	No	No	No	No	No	No	No	No	Yes	11% (1/9)
Principle 6: Human-centered application of AI	1. Guaranteeing human interaction in health services	No	No	No	No	No	No	No	No	No	0% (0/9)
	2. Ensuring human oversight throughout the AI lifecycle	No	No	No	No	No	No	No	No	No	0% (0/9)
	3. Clinical consent management	No	No	No	No	No	No	No	No	No	0% (0/9)
Research Bias	1. Real-world data application	Yes	Yes	Yes	Yes	Yes	Yes	No	Yes	Yes	89% (8/9)
	2. Diverse backgrounds	Yes	Yes	Yes	Yes	Yes	Yes	Yes	Yes	Yes	100% (9/9)
	3. Funding and conflicts of interest	Yes	Yes	Yes	Yes	Yes	Yes	Yes	Yes	Yes	100% (9/9)
Provider Expertise Bias	1. Provider bias consideration	No	No	No	No	No	No	No	No	No	0% (0/9)
	2. Consistency of clinical guidelines	No	No	No	No	No	No	No	No	No	0% (0/9)
Embedded Data Bias	1. Data collection bias analysis	Yes	No	No	Yes	No	No	No	No	No	22% (2/9)
	2. Synthetic data integration	No	Yes	No	Yes	NA	Yes	Yes	No	Yes	63% (5/8)
	3. Missing or incomplete data management	No	Yes	No	No	NA	No	No	Yes	No	25% (2/8)
Environmental and Life-Course Bias	1. Environmental and life factors impact	Yes	No	No	No	No	No	No	No	No	11% (1/9)
Empathy or Contextual Bias	2. Knowledge of cultural or procedural factors of the data	No	No	No	No	No	No	No	No	No	0% (0/9)
Implicit bias	1. Pre-existing biases in data	No	No	No	No	No	No	No	No	No	0% (0/9)
	2. Worse clinical outcomes in vulnerable groups	No	No	No	No	No	No	No	No	No	0% (0/9)
Selection Bias	1. Population representativeness assessment	Yes	Yes	No	No	No	No	Yes	Yes	Yes	56% (5/9)
	2. Participant diversity and data during the training phase	No	Yes	No	No	No	No	No	Yes	No	22% (2/9)
	3. Sampling bias assessment	Yes	Yes	Yes	Yes	Yes	Yes	No	No	No	67% (6/9)
Measurement bias	1. Inaccuracies in data collection	No	Yes	No	No	NA	No	No	Yes	No	25% (2/8)
	2. Standardization of data collection	No	No	No	Yes	No	No	No	No	No	11% (1/9)
	3. Data biases affecting AI model performance	No	No	No	No	No	No	No	No	No	0% (0/9)
Confounding Bias	1. Confounding factors analysis	Yes	Yes	Yes	No	Yes	Yes	No	Yes	No	67% (6/9)
Algorithmic Bias	1. Performance indicators reporting	Yes	Yes	Yes	Yes	Yes	Yes	Yes	Yes	Yes	100% (9/9)
	2. Statistical assumptions check	No	No	Yes	No	No	No	No	No	No	11% (1/9)
Temporal Bias	1. Temporal changes impact	No	No	No	No	No	No	No	No	No	0% (0/9)
	2. Adjustments for temporal changes	NA	NA	NA	Yes	NA	NA	NA	NA	NA	100% (1/1)

Note: Yes = The criterion is met. No = The criterion is not reported, not met, the information is unclear, or the criterion is partially reported. Not applicable (NA) = The criterion does not apply to the study being evaluated and should not be considered.

**Table 4:** Number of total citations, citations mean by year, and citations of replication/use of the AI model.

Reference	Total Citations	Mean by Year	Replicate or Use the AI Model
[26]	3	3.0	0
[27]	2	1.0	0
[21]	1	0.5	0
[24]	0	0.0	0
[25]	3	1.5	0
[28]	10	3.3	3
[29]	7	1.8	0
[22]	31	7.8	0
[23]	71	10.1	1

Note: Search date 4 November 2024. Source: Google Scholar.

## Data Availability

Not applicable.

## Supplementary Materials

The following supporting information can be downloaded at https://www.mdpi.com/article/10.3390/cancers17030407/s1, Table S1: Bias assessment criteria for AI models. Table S2: Reasons for exclusion during full-text review (*n* = 6). Section S1: Detailed description of the characteristics of the studies included, by stage. Table S3: Levels of AI performance metrics. Table S4: PROBAST risk of bias. Table S5: Linear regression analysis of AI performance metrics (exposure) and the number of citations in google scholar (outcome).
