# Supplementary material for: Exploring Artificial Intelligence Biases in Predictive Models for Cancer Diagnosis
Source: Cancers (Basel). 2025 Jan 26;17(3):407. doi: 10.3390/cancers17030407 (PMC11816222; doi:10.3390/cancers17030407)
Supplement: Supplementary file 1 [file cancers-17-00407-s001.zip › cancers-3382576-supplementary.pdf]

Supplementary Materials

Supplementary Material Table S1. Bias assessment criteria for AI models.

Bias Criterion	Items	Yes/No Quantification Questions
Principle 1: Transparency	1. Data and procedure access of the training	1. Did the researchers provide access to the data and processes used to create the model during training?
	2. Data and procedure access of the testing	2. Did the researchers provide access to the data and processes used to create the model during testing?
	3. Reproducibility materials access	3. Did the investigators provide access to all materials necessary to ensure the reproducibility of the study (i.e., code, data, hyperparameters, procedures)?
Principle 2: Informed Stakeholders	1. Professional training in AI usage	1. Were health professionals trained on how to use the AI model for making clinical decisions?
	2. Patient's informed consent for the use of data in AI.	2. Were patients informed that their data was used to train the AI model for clinical decisions?
Principle 3: Fairness and Justice	1. Model fairness measures	1. Are there measures in place to assess the fairness of the model (e.g., equal treatment across different demographic groups, reporting of AI performance metrics between groups)?
	2. Diversity of participants included is reported	2. Does the paper analyze the diversity of participants included in the model evaluation (e.g., race, gender, or age in training, testing, and validation)?
	3. Compliance with specific ethical guidelines for AI models	3. Does the paper mention the use of specific ethical guidelines for the AI model?
Principle 4: Accountability	1. Compliance with legal and regulatory requirements	1. Does the paper report that the AI model evaluated meets the legal, regulatory or institutional requirements governing the use of health data?
	2. Adherence to ethical standards	2. Does the paper mention that the evaluated AI model has undergone an ethical review to approve data usage?
	3. Statement of responsibility	3. Does the paper specify who assumes legal, regulatory, and ethical responsibility for the use and decisions of the AI model (e.g., researchers, healthcare workers, institutions)?
Principle 5: Oversight and privacy	1. Patient data privacy protection	1. Do the researchers explain how patient data privacy and confidentiality were maintained?
	2. Use of privacy-enhancing technologies	2. Do the researchers mention the use of privacy-enhancing technologies to ensure data sharing was conducted privately and confidentially?
	3. Ensuring the autonomy of health professionals and patients	3. Does the paper report that the AI model safeguards the autonomy of healthcare professionals and patients in clinical decision-making?
Principle 6: Human-centered application of AI	1. Guaranteeing human interaction in health services	1. Does the paper report that the AI model does not replace or eliminate human interaction in healthcare decision-making?
	2. Ensuring human oversight throughout the AI lifecycle	2. Does the paper address human involvement throughout different phases of the AI model's lifecycle?
	3. Clinical consent management	3. Does the paper report that there was verification, supervision, acknowledgment, and final approval of the healthcare professional's consent before any clinical decision?
Research Bias	1. Real-world data application	1. Does the paper report that the study used real-world data to improve the training or testing of the AI model?
	2. Diverse backgrounds	2. Does the paper report that the research team has a diverse background?
	3. Funding and conflict of interest	3. Does the paper address whether funding, conflicts of interest, or political decisions influenced the research?
Provider Expertise Bias	1. Provider bias consideration	1. Does the paper consider potential biases, inconsistencies, or stereotypes from healthcare providers that might affect the data or how the AI model is applied?
	2. Consistency of clinical guidelines	2. Does the paper report that the clinical guidelines used for care and data collection were consistent for all participants?
Embedded Data Bias	1. Data collection bias analysis	1. Have biases in the data or the data collection process been analyzed?

	2. Synthetic data integration	2. Does the paper address the integration of synthetic or imputed data into the AI model?
	3. Missing or incomplete data management	3. Does the paper explain how missing or incomplete data is managed to avoid bias?
Environmental and Life-Course Bias	1. Environmental and life factors impact	1. Does the paper consider how environmental, occupational, or life factors could influence the model's outcomes?
Empathy or Contextual Bias	2. Knowledge of cultural or procedural factors of the data	2. Do the authors demonstrate an understanding of the people, culture, and places represented in the data used for the AI model?
Implicit bias	1. Pre-existing biases in data	1. Does the paper discuss the presence of preexisting biases, such as stereotypes or flawed assumptions, in the data used?
	2. Worse clinical outcomes in vulnerable groups	2. Does the paper address whether implicit bias is associated with negative clinical interactions and worse treatment outcomes for vulnerable patients?
Selection Bias	1. Population representativeness assessment	1. Does the paper evaluate if the data used to train the AI model represents the target population?
	2. Participant diversity and data during the training phase	2. Does the paper mention if the model was trained on data that includes diverse demographic groups and health conditions?
	3. Sampling bias assessment	3. Does the paper assess whether sampling methods might have introduced bias, leading to certain groups being overrepresented or underrepresented?
Measurement bias	1. Inaccuracies in data collection	1. Does the paper evaluate whether there were inaccuracies or incomplete data entries during the data collection process by clinicians or clinical devices?
	2. Standardization of data collection	2. Does the paper discuss whether measures were taken to mitigate measurement bias and ensure consistency among all participants (e.g., use of guidelines or equipment calibration)?
	3. Data biases affecting AI model performance	3. Does the paper evaluate how inaccuracies in clinical device data might affect the AI model's outcomes?
Confounding Bias	1. Confounding factors analysis	1. Does the paper explore if there are any confounding factors (e.g., socioeconomic status) that could influence both health outcomes and AI predictions?
Algorithmic Bias	1. Performance indicators reporting	1. Does the paper clearly evaluate the key performance indicators of the AI system?
	2. Statistical assumptions check	2. Does the study mention if the data meets the assumptions of the statistical analyses used?
Temporal Bias	1. Temporal changes impact	1. Does the paper discuss how changes over time (e.g., new guidelines, changes in data collection) could have affected the AI model's predictions?
	2. Adjustments for temporal changes	2. Are adjustments made to account for changes over time, such as changes in data distribution or video sequences?

Supplementary material Table S2. Reasons for exclusion during full-text review (n=6).

Title	Year	DOI	Reason for exclusion
Potential Role of Generative Adversarial Networks in Enhancing Brain Tumors.	2024	10.1200/CCI.23.00266	Wrong outcome, the focus is on improving image quality rather than performing diagnosis or screening.
Prostate Cancer Risk Stratification by Digital Histopathology and Deep Learning.	2024	10.1200/CCI.23.00184	Wrong outcome, the study focuses on prognosis rather than diagnosis.
MOSAIC: An Artificial Intelligence-Based Framework for Multimodal Analysis, Classification, and Personalized Prognostic Assessment in Rare Cancers.	2024	10.1200/CCI.24.00008	Wrong outcome, the focus is on AI for prognosis and classification.
Natural Language Processing-Assisted Classification Models to Confirm Monoclonal Gammopathy of Undetermined Significance and Progression in Veterans' Electronic Health Records.	2023	10.1200/CCI.23.00081	Wrong outcome, the study focuses on AI for extracting data from electronic health records (EHR).
Using Adversarial Images to Assess the Robustness of Deep Learning Models Trained on Diagnostic Images in Oncology.	2022	10.1200/CCI.21.00170	Wrong outcome, the study assesses the stability of AI.
MLCD: A Unified Software Package for Cancer Diagnosis.	2020	10.1200/CCI.19.00129	Wrong outcome, the study describes an existing tool instead of developing or validating an AI model.

S1. Detailed description of the characteristics of the studies included, by stage.

Training phase

Regarding the studies that reported participant information (7/9), an average of 11,034 participants were included per study (range: 103 to 56,474) during the training phase of the AI model. It is noteworthy that only two studies had sample sizes exceeding ten thousand participants [12,17], while the remaining studies had sample sizes below 900 participants.

Six reported participant gender [12,15–19] (6/7), and one study failing to specify the proportion of male and female participants [13] (1/7). One study of these studies exclusively included female participants, as it focused on ovarian cancer [18]. Additionally, one study included both males and females but reported a significantly higher proportion of males (98%) during the training phase of the AI model, potentially leading to biased results that may not be generalizable to females [19]. The other four studies included both males and females, with male proportions ranging from 60% to 35% [12,15–17].

The median age across the seven studies reporting participant characteristics ranged from 51.3 to 73 years. One study predominantly included elderly males (median age 73 years) during the AI model training phase, which may limit the generalizability of the findings to younger adults [19].

Only six studies reported both the gender and number of participants with and without cancer [12,15–19] (6/7), while five studies reported the mean age of participants with and without cancer [12,15–17,19] (5/7). The number of participants with cancer ranged from 26 to 3,322, whereas the number of participants without cancer ranged from 77 to 53,152.

Testing phase

Of the five studies that conducted a testing phase, two reported using 30% of the entire dataset during testing but did not describe the characteristics of the included participants [12,13] (2/5).

Three studies provided detailed information on participant characteristics [15,17,18] (3/5), which included a total of 168 to 3,798 participants. The proportion of males ranged from 0% in a study focused solely on ovarian cancer to 47% or 35% in studies on pancreatic cancer. The median age across these studies varied from 50.8 to 62 years. In terms of the proportion of patients with and without cancer, the studies included between 30 and 2,127 participants with cancer and between 138 and 1,671 participants without cancer.

Validation phase

Of the four studies that conducted a validation phase, only two studies reported detailed descriptive characteristics of the included participants [15,18] (2/4). Among these, one study included 2,000 female participants with a median age of 47.5 years, of whom only 4.9% (n=98) had cancer [18]. The other study included 186 participants, 47% of whom were male, with a median age of 62 years, and 39.2% of the participants had cancer (n=73) [15].

On the other hand, of the two remaining studies that conducted a validation phase, one reported using 10% of the dataset for validation but did not provide specific participant characteristics [13], and the other provided information on frames or images from 68 cases but did not describe participant characteristics in detail [14].

Supplementary material Table S3. Levels of AI performance metrics.

Level	Sensitivity	Specificity	Accuracy	Precision	F1	ROC/AUC
1.00-0.90	30% (3/10)	33% (3/9)	50% (3/6)	17% (1/6)	25% (1/4)	55% (6/11)
0.89-0.80	30% (3/10)	44% (4/9)	50% (3/6)	0% (0/6)	25% (1/4)	36% (4/11)
<0.79	40% (4/10)	22% (2/9)	0% (0/6)	83% (5/6)	50% (2/4)	9% (1/11)

Note: Although only 9 studies were included, some studies reported two or more AI performance metrics across the training, testing, or validation phases. Therefore, the denominator may exceed the number of included studies. Because not all studies report the different AI performance metrics, the denominator is different for each metric.

Supplementary material Table S4. PROBAST risk of bias.

	ROB				Applicability			Overall	
**Study**	**Participants**	**Predictors**	**Outcome**	**Analysis**	**Participants**	**Predictors**	**Outcome**	**ROB**	**Applicability**
Johnson (2024)	+	+	+	+	+	+	+	+	+
Matchaba (2023)	+	+	+	+	+	+	+	+	+
Bojesen (2023)	-	+	+	?	-	+	+	?	+
Eminaga (2023)	-	+	+	?	-	+	+	?	+
Firpo (2023)	+	+	+	?	+	+	+	?	+
Reilly (2022)	+	+	+	+	+	+	+	+	+
Shah (2021)	+	+	+	+	+	+	+	+	+
Chen (2021)	?	+	+	+	?	+	+	+	?
Eminaga (2018)	-	+	+	+	-	+	+	+	-

Note: ROB = risk of bias; + indicates low ROB/low concern regarding applicability; - indicates high ROB/high concern regarding applicability; and ? indicates unclear ROB/unclear concern regarding applicability.

Supplementary material Table S5. Linear regression analysis of AI performance metrics (exposure) and the number of citations in google scholar (outcome).

Variable	Coefficient	p
Sensitivity	2.08	0.818
Specificity	-13.31	0.590
Accuracy	0.00	0.949
Precision	6.88	0.316
F1	0.00	Not reported
ROC/AUC	9.92	0.802

Note: Model adjusted for year of publication. Date of extraction of citations in google scholar on November 4, 2024. Metrics were excluded if it was unclear to which phase they belonged (training, testing and validation).
